# In Vitro Generation of Oocytes from Ovarian Stem Cells (OSCs): In Search of Major Evidence

**DOI:** 10.3390/ijms20246225

**Published:** 2019-12-10

**Authors:** Erica Silvestris, Stella D’Oronzo, Paola Cafforio, Anila Kardhashi, Miriam Dellino, Gennaro Cormio

**Affiliations:** 1 Gynecologic Oncology Unit, National Cancer Center, IRCCS Istituto Tumori “Giovanni Paolo II”, 70124 Bari, Italy; a.kardhashi@oncologico.bari.it (A.K.); miriamdellino@hotmail.it (M.D.); gennaro.cormio@uniba.it (G.C.); 2 Department of Biomedical Sciences and Human Oncology, Section of Internal Medicine and Clinical Oncology, University of Bari Aldo Moro, 70124 Bari, Italy; ester86d@gmail.com (S.D.); paola.cafforio@uniba.it (P.C.); 3 National Cancer Center, IRCCS Istituto Tumori “Giovanni Paolo II”, 70124 Bari, Italy; 4 Department of Biomedical Sciences and Human Oncology, Unit of Obstetrics and Gynecology, University of Bari Aldo Moro, 70124 Bari, Italy

**Keywords:** Ddx4, ovarian stem cells, fertility preservation, ovarian failure, anti-cancer treatments

## Abstract

The existence of ovarian stem cells (OSCs) in women as well as their physiological role in post-menopausal age are disputed. However, accumulating evidence demonstrated that, besides the animal models including primarily mice, even in adult women putative OSCs obtained from ovarian cortex are capable to differentiate in vitro into oocyte-like cells (OLCs) expressing molecular markers typical of terminal stage of oogonial cell lineage. Recent studies describe that, similarly to mature oocytes, the OSC-derived OLCs also contain haploid karyotype. As proof of concept of their stem commitment, OSCs from mice differentiated to oocytes in vitro are suitable to be fertilized and implanted in sterilized animals resulting in embryo development. Despite enthusiasm for these data, which definitely require extended confirmation before considering potential application in humans for treatment of ovarian insufficiency, OSCs appear suitable for other clinical uses, restoring the endocrine derangements in premature ovarian failure or for fertility preservation in oncologic patients after anti-cancer treatments. In this context, the selection of viable oocytes generated from OSCs before chemotherapy protocols would overcome the potential adjunct oncogenic risk in women bearing hormone-dependent tumors who are repeatedly stimulated with high dose estrogens to induce oocyte maturation for their egg recruitment and cryopreservation.

## 1. Introduction

The detection and isolation of ovarian stem cells (OSCs) from mouse and human ovaries have recently induced both enthusiasm and disbelief in the field of reproductive medicine as well as in stem cell biology and translation to clinical medicine. This discovery also appears contrary to the well accepted dogma that mammalian females are endowed with a fixed number of oocytes and follicles at birth, which undergo depletion with age in parallel with a sudden exhaustion of follicles resulting in anovulation and menopause. However, after the initial challenge to this decade-old dogma, concurrent studies now strongly suggest that ovaries of mammals generate new oocytes and follicles during their biologic life.

Two distinctive opinions concerning oogenesis were raised in the last 50 years, first by Waldeyer and his group who claimed that before and after birth, oocytes originate from ovarian germinal epithelium [1], whereas Beard and coworkers proposed that all oocytes are generated during the embryonic time before the birth and thus are exhaustively utilized until menopause [2].

Subsequently, other investigators described the existence of OSCs in the adult mammalian ovary that are expected to undergo neo-oogenesis and differentiate into OLCs either spontaneously or under proper culture conditions [3,4]. Moreover, animal models of infertility by iatrogen depletion of the ovarian reserve proved the efficacy of OSCs in experimental re-fertilization [5]. Despite these data, an eclectic skepticism persists, and the consensus on the OSCs existence in adult ovaries supporting their putative in vivo role for the postulated neo-oogenesis and follicle assembly needs to be expanded.

Here, we revisit the literature with respect to the occurrence of OSCs in the woman ovaries, also based on our experimental work, to address concurrent controversies.

## 2. OSCs: Do They Really Exist?

Zuckerman et al., in 1951, showed a preliminary observation of the OSCs [6]. They acknowledged a long-held dogma declaring that in postnatal mammalian ovaries of most species no renewable germinal OSCs may exist, thus sustaining the theory that a fixed pool of oocytes is committed during the life for the female fertility with a progressive aging-related decline until the complete exhaustion in menopause.

This assumption was subsequently rebutted by Tilly and co-workers [5], who investigated in both young and adult murine ovaries the existence of OSCs capable to guarantee the bioavailability of oocyte and follicles after birth. In their trials, to investigate the fate of GFP (green fluorescent protein) positive oocytes in wild-type grafts, they transplanted these animals, previously sterilized by busulfan, with ovarian fragments conjugated with the GFP from adult wild type mice. Nevertheless, along with the persistence of these cells, the regenerated granulosa cells close to the GFP-positive oocytes in the transplanted pieces were GFP-negative. This result suggested that OSCs after migrating into the transplanted tissue were able to regenerate new follicles in adult mice. The skepticism also regarded the methods used to distinguish unhealthy from healthy follicles as well as the busulfan adopted to induce sterility, whose effects on OSCs are indeed unknown [5].

The debate, however, continued when Tilly and colleagues speculated about the existence of a putative reservoir of oogonial stem cells OSCs in bone marrow (BM) of adult mice. In this regard, they first described the presence of several germ cell line markers as SSEA-4 (stage specific embryonic antigen 4), OCT4 (octamer binding transcription factor 4), MVH (mouse vasa homologue), STELLA, DAZL and FRAGILIS in BM from adult female mice and then transferred their BM-derived OSCs into adult females pre-treated with both busulfan and cyclophosphamide. As result, they observed a consistent generation of new oocyte [7].

Subsequently, other investigators from the Virant-Klun’s group reported the presence of small, SSEA-4^+^ stem cells of 3–5 μm of diameter in human ovarian superficial epithelium (OSE) that abundantly expressed markers of primordial and pluripotent germ cells as well as the property to independently differentiate into oocytes-like structures in vitro [8]. To isolate the OSCs from the ovarian epithelium, initial methods used mechanical scraping and subsequent enzymatic digestion of ovarian cortex fragments [9]. The putative OSCs were thus separated by density gradient centrifugation and grown in vitro to verify their differentiation into oocyte-like cells. However, based on their lack of SYCP3 (synaptonemal protein complex 3), a meiosis marker, these cells were proposed as adult OSCs expressing markers of the embryonic stem cell lineage [10]. Subsequent approaches were based on both immunomagnetic and fluorescent cell sorting (FACS) to detect both germ-line markers as DEAD (Asp-Glu-Ala-Asp)-box polypeptide-4 (Ddx4), FRAGILIS, and STELLA, as well as stemness molecules including OCT4A and SSEA-4, and confirmed the superiority of this method for OSC enrichment in the sorted population with respect to oocytes or contaminating cells from other lineages. However, although Ddx4 is a 79.3 kDa protein with an extracellular COOH-domain and a cytoplasmic tale exclusively expressed by both oogonial and spermatogonial cells, several criticisms required that OSCs need to be purified only by positive selection of Ddx4^+^ cells, since this protein is prevalently cytoplasmic in early steps of oocyte differentiation.

Therefore, Tilly and co-workers standardized a FACS-based protocol aimed at isolation of OSCs by linking the cell surface portion of Ddx4, namely, through targeting its COOH terminus [3]. The cells obtained by this technique were thus found to differentiate in vitro to mature oocytes, enlarged up to 30–50 µm of diameter, and acquire markers of terminal differentiation as GDF-9 (growth differentiation factor 9) and NOBOX (newborn ovary homebox), as well as patterns of meiosis with specific markers and molecular cell modifications supporting the haploid karyotype. Besides the controversy concerning the Ddx4 as cytoplasmic and/or membrane antigen expressed by OSCs in their maturation, Ddx4 positive cells isolated from postmenopausal women were subsequently proved to differentiate in vitro and gain the pattern of large oocyte like cells showing morphology similar to mature oocytes [11].

**Figure 1:**
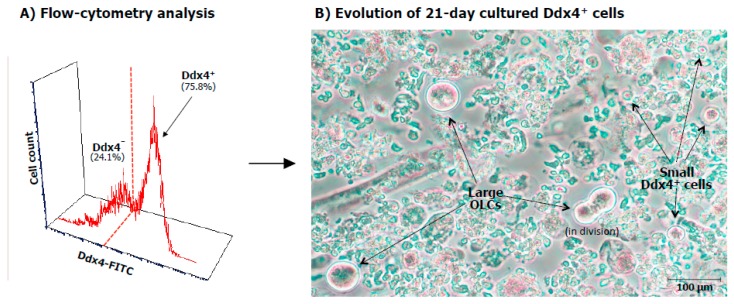
(**A**) FACS analysis of OSCs. Fluorescence flow cytometry analysis of OSCs after separation by immunomagnetic procedure. As shown, the Ddx4^+^ cell population was expanded after selection since the Ddx4^+^ cells were up to 75.8% of the full cortical ovarian suspension, with a remarkable fluorescence intensity, thus suggesting the high molecular expression of Ddx4 molecule. (**B**) In vitro differentiation to oocyte-like cells (OLCs). After 21 days of culture, small Ddx4^+^ cells differentiated in large OLCs reaching up to 75–80 μm in size, with prominent nuclei and a perinuclear accumulation of organelles. At the same time, several small Ddx4^+^ cells maintained their size in cultures together with the enlarged ones.

Initial work from our group binding the Ddx4 molecule for immunomagnetic selection of ovarian cortex cell suspension, showed that this protein is abundantly present in cytoplasm and at lower intensity on the cell membrane of three weeks cultured OSCs, thus confirming that such a peculiar stemness marker is expressed during oocyte maturation and that by using reagents binding its extracellular domain, it is possible to isolate the cells exposing the original germ-line marker belonging to OSCs [12]. Figure 1A depicts the enrichment of Ddx4^+^ cell population by FACS analysis in the full cell suspension obtained from the ovarian cortex and (B) the differentiation of these cells after 21 days of culture. As can be seen, although in the presence of Ddx4^+^ cells acquired the morphology and size of OLCs, other small Ddx4^+^ cells maintained their viability in the shape of small cells.

**Figure 2:**
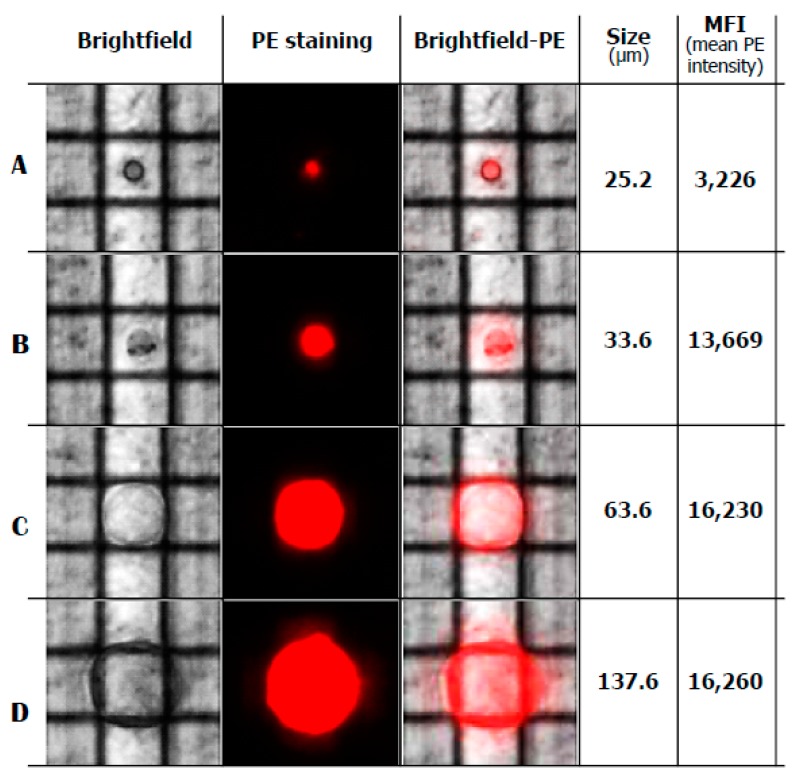
Selection and isolation of single cells by DEPArray technology. Image of single, trapped small (**A**), intermediate (**B**) and large (**C**,**D**) cells after 21-day culture of Ddx4^+^ cells. Cells moved under dielectrophoretic force, were entrapped within the cartridge cages, selected by size and mean fluorescence intensity to PKH26 red (PE, phycoerythrin fluorescence) and isolated for further molecular analysis.

We also isolated both large and small Ddx4^+^ cells by the DEPArray separation [13] using the dielectric field movimentation for single cell purification. As shown in Figure 2, Ddx4^+^ cells of different size from 25.2 up to 137.6 µm were isolated by this technology and all of them expressed variable levels of Ddx4-related fluorescence, as mean fluorescence intensity (MFI) which was dependent on the cell dimensions. This analysis confirmed that only a minority of Ddx4^+^ cells are capable of enlarging and acquiring the shape of OLCs, whereas other cells maintained their small size thus supporting the hypothesis of their asymmetric division for self-renewing and next expansion [14].

## 3. Differentiation of OSCs in Oocytes: Hypothesis or Reality?

Recently, following the successful isolation of OSCs, several investigators have independently experienced their survival, differentiation and growth in culturing systems. In fact, ovarian epithelial cells from both non-menopausal and menopausal women have been shown to generate in vitro apparently mature OLCs, thus postulating that the neo-oogenesis may occur in the ovary senescence [9].

By using their FACS-based protocol, Tilly and co-workers observed that isolated OSCs differentiated in vitro to large mature oocytes, with progressive enlargement up to 30–50 μm in diameter, and expressed terminal markers as zona pellucida (ZP) glycoproteins, GDF-9, NOBOX, YBX2 (Y-box binding protein 2), SYCP3 and, moreover, molecular modifications toward the haploid karyotype [15].

Horan and Williams [16] also discussed the historical perspective of OSCs and examined the controversies regarding selection of markers as Ddx4 to isolate OSCs by flow cytometry, asking whether or not OSCs comparably exist across various species. However, the controversies surrounding the existence of OSCs were improperly related to the technical procedures for their isolation since their detection also in human adult ovaries was suggested by growing studies. They debated that Tilly’s group [17] focused the bigger sized (5–8 μm) OSCs, whereas the Virant-Klun’s group [18] considered the smaller sized (2–4 μm) pluripotent stem cells as very small embryonic-like stem cells (VSELs) located within the ovary surface epithelium (OSE). Therefore, based on both size and marker expression of these cells in human OSE, as well as in other animal species, they concluded that there are two distinct populations of stem cells, namely, the small sized, pluripotent VSELs (expressing nuclear OCT-4A), and the larger OSCs (with cytoplasmic OCT-4B) [19].

VSELs are epiblast-derived pluripotent stem cells corresponding to primordial germ cells that persist in very small numbers in adult gonads [20], whereas OSCs appear as tissue-specific progenitors with larger size and dissimilar gene expression with respect to pluripotent VSELs. To this regard, novel data suggest that tissue-specific progenitors deriving from asymmetric cell division of VSELs, may undergo symmetric cell divisions and subsequent clonal expansion including the sphere formation before their differentiation into tissue specific cell lineage, as occurs for sperms in testis and blood cells in BM [21]. However, the role in vivo of these stem cells in adult mammalian ovaries and the intrinsic factors that regulate their biology are still unclear. Bhartiya and co-workers postulated that VSELs are the most primitive, pluripotent stem cells in ovary that give rise to committed tissue-specific progenitors including OSCs, expressing OCT-4 in cytoplasm in association to other germ cell markers [22]. It has been also reported that VSELs are relatively quiescent in vitro, in contrast with OSCs that rapidly undergo division and generate germ cell nests before their differentiation to oocytes.

In line with studies by others, we have recently reported that OSCs express receptors for the follicle stimulating hormone (FSH) and that the process of asymmetric cell division including the self-renewal and generation of OSCs by VSELs appears to be influenced by FSH, particularly through the alternatively spliced isoform (FSHR3) of the growth factor type 1 receptor [23]. We have also provided evidence that OSCs are obtainable from menopausal women, thus suggesting that stem-like cells with ovarian germline pattern are definitely detectable during the entire ovary life. By using immunomagnetic separation based on the expression of the membrane Ddx4 followed by DEPArray, culture-derived OLCs of large size and showing markers of mature oocytes were acquired from fertile as well as from menopausal women [13]. Ovarian cortex portions were treated by immunomagnetic sorting using a rabbit anti-human Ddx4 antibody, and the sorted populations were evaluated in their Fragilis and SSEA-4 expression. Using this procedure, we isolated Ddx4^+^OSCs from 19 menopausal and 13 non-menopausal that were cultured up to 21 days. After three weeks, large and small cells were individually isolated and typed for both early and late differentiation markers. Large OLCs were then isolated by the DEPArray sorter, and SYCP3 and GDF-9 as terminal germline markers, in combination with DPPA3 (developmental pluripotency-associated protein 3) as primordial marker, were quantified by droplet digital PCR [13].

**Figure 3:**
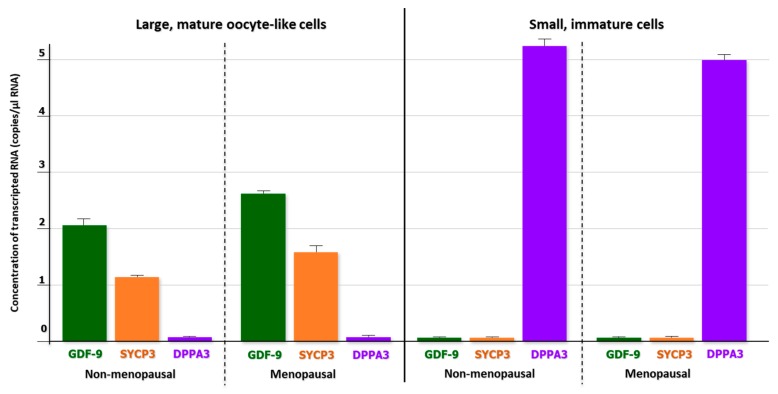
Quantitative evaluation (copies/μL) of gene expression in large OLCs and small cells, both Ddx4^+^ (QuantaSoft Software). In large OLCs from the non-menopausal and menopausal samples, 2 and 2.6 copies/μL of growth differentiation factor (GDF-9) and 1.2 and 1.5 copies/μL of synaptoneal complex protein 3 (SYCP3), respectively, were detected. By contrast, in small, immature cells from both non-menopausal and menopausal women, no expression of GDF-9 and SYCP3 was found, whereas 5.1 and 5.0 copies/μL of the developmental pluripotency-associated protein 3 (DPPA3) gene were identified. This marker is predominantly expressed in early differentiation stages of the oogonial cell lineage. These results suggested that large OLCs were molecularly associable to mature oocytes, whereas the small cells remained in their primordial differentiation stage of stem cells.

Despite the lower number of Dxd4^+^ cells including both Fragilis^+^ and SSEA-4^+^ fractions from menopausal women with respect to the non-menopausal cohort, no differences were observed in the RNA levels of both GDF-9 and SYCP3 in large cells, in contrast with undetectable amounts of DPPA3 mRNA, supporting the condition of mature cells along their own differentiative pathway (Figure 3).

To assess their final differentiation to OLCs, we explored the ploidy content by FISH (fluorescence in situ hybridization) with probes for chromosomes X and 5. A single fluorescent signal was detected in these large Ddx4^+^ differentiated cells, thus supporting their haploid state of OLCs [13].

Our work, in junction with those from others, supports the terminal differentiation of OSCs from the ovarian surface of both fertile and post-menopausal women to mature OLCs

## 4. Novel Evidence Suggesting the Existence of OSCs

Despite a diffuse enthusiasm for these findings, in particular for the acquired information on the real existence of OSCs, a few skepticism sometimes arises with respect to question whether or not these cells are suitable to improve oogenesis resulting in prolongation and restoration of the fertility potential in women [24].

To this regard, more recently, further evidence supported once again the occurrence of a postnatal oogenesis from mammal germ cells [7]. In fact, in a recent study by Akahori and Colleagues, it has been also hypothesized that the differentiation of OSCs to competent oocytes is directly supported by somatic cells surrounding their location in cortical niches [25]. Indeed, undifferentiated granulosa cells (UGCs), namely, defined cells which physiologically proliferate together with oocytes within the developing follicles, regulate their final maturation resulting in the expression of stemness genes and related markers [26]. By using the FACS sorting, the authors isolated a somatic ovarian cell population expressing several stem cell associated genes as Pou5f1 (POU domain class-5 transcription factor-1) and Nanog. These cells were subsequently expanded in vitro and showed typical UGC pattern including the expression of both Foxl2 (Forkhead box L2) and Wnt4 (Wingless-type MMTV integration site family member 4) genes, as well as FSH-R (receptor), thus confirming their potential role in sustaining the postnatal oogenesis [27]. In this context, it has also been postulated that UGCs would hypothetically derive from pluripotent stem cells (PSC) and can thus be isolated and successfully expanded in vitro to support the OSC differentiation.

These findings in woman OSCs appear in line with previous data in animal models. By culturing mouse embryonic stem cells (ESCs), Hübner and his team observed several follicle-like structures capable of steroidogenesis and support the extrusion of OLCs, which were referred to the UGC [28]. Subsequently, Woods et al. described the first study of UGCs adopting a dual-reporter mouse ESC line engineered to shown both EGFP (enhanced green fluorescence protein) under the Pou5f1 gene promoter, and DsRed (red fluorescent protein) under the Foxl2 control gene promoter, respectively, with the aim to simultaneously observe the germ and granulosa cell development. Finally, they detected in ESC cultures, the formation of follicle-like structures with EGFP-positive germ cells, namely OLCs, enclosed within DsRed-positive somatic cells or pre-granulosa cells. When the DsRed-positive cells were isolated by FACS and analyzed, a gene expression profile including Foxl2, Wnt4, Follistatin typical of the UGC phenotype was showed. However, by extending the culture time, these cells underwent a defined granulosa cell phenotype although at an early stage of differentiation with the activation of FSH-R, AMH (anti-Müllerian hormone) and STAR (steroidogenic acute regulatory protein) gene expression [29].

Thus, in search of a model improving OSC differentiation to mature oocytes, it is conceivable that patient-derived induced-PSCs may be useful and putatively usable to generate autologous granulosa cells to prompt OSC maturation and generation of new, mature oocytes [30]. To this purpose, other investigators have recently suggested adopting patient-matched autologous mitochondria from ovarian stem cells to evaluate the functional properties in oocytes [31], since incorporation of appropriate somatic cell partners could permit their differentiation into fully functional eggs [32].

These results in the OSC basic investigative studies definitely support their existence and thus open new avenues for future studies in the woman infertility field. On the other hand, the ovarian stemness research parallels the data obtained in the testis stemness advancement. Recently, Hermann et al. described the capability of spermatogenic stem cells from thawed testis to restore spermatogenesis once transplanted in the gonad niche [33], while a similar approach was attempted by Sato and co-workers to promote spermatogenesis outside the body. In fact, they cultured in vitro immature mouse testis obtaining spermatids that were intracytoplasmicly injected in oocytes resulting in fertile offspring in rhesus monkey [34]. Therefore, such encouraging findings open new possibilities for future adoption of OSC-based technologies to revert human ovary insufficiency.

## 5. OSC-Derived Oocytes: The Lesson from Animal Models

The knowledge improvement on the OSC topic was also advantaged by investigation in animal models. In fact, particularly mice have been studied to explore the role and the potential applications of these cells and by applying modern procedures of molecular medicine, it has been possible to generate transgenic mice in which OSCs are fluorescently marked and easily identified in their evolution [35].

However, other species have also been used in studying postnatal oogenesis and Drosophila as well as other invertebrates have been proved to generate new OLCs in adult life, but these animal models lack a biologic similarity to allow direct comparison with mammals [36]. Moreover, adult pigs have been investigated by Bui and co-workers who installed cultures of ovarian biopsies in which OSCs expressing stemness markers were detected [37]. However, despite these proofs of existence of neo-oogenesis in different animal species, Telfer and his group asserted that this evidence did not prove the subsistence of a functional neo-oogenesis in adult human ovaries [38,39].

Thus, White and co-workers provided evidence of the existence of a cell population in both adult mouse and human ovaries, that can be expanded in vitro forming oocyte-like structures and expressing specific biomarkers as Ddx4, KIT (kit oncogene), LHX8 (LIM homeobox protein 8). To this regard, the authors, not only optimized the collection of these cells but also revealed that under suitable conditions both in vitro and in vivo, the OLCs were capable of being fertilized and undergoing embryo development [11]. To prove these results, in their work, White and co-workers, first transfected putative murine OSCs with a lineage marker expressing GFP, and then 5–6 months after the injection into adult mice ovaries, showed that GFP-expressing oocytes could be recovered from newly ovulated mice. The retrieved oocytes were thus subjected to in vitro fertilization and embryo culture. From 5 mice, 31 cumulus-oocyte complexes were recovered, and 8 out of them successfully owned the GFP-expressing lineage marker in oocytes. Notably, from each of the 5 mice cohort, at least one GFP-positive oocyte was retrieved, while all developmental stages up to the blastocyst formation were demonstrated to bear GFP fluorescence [11].

Similarly, the authors isolated and transfected human OSCs from ovarian cortical strips of adult women. The GFP-expressing OSCs were inoculated into human cortical strips and subsequently transplanted into nonobese diabetic (NOD)-severe combined immunodeficiency (SCID) mice, with the detection after 7–14 days of a pool of oocytes surrounded by somatic cells expressing meiotic markers [11].

These results provide a definitive trend of evidence on the function of OSCs in generating OLCs in relation to the expression of the YBX2, as well as other oocyte-specific lineage markers as Ddx4, KIT and LHX8, and counteract indeed the previous skepticism concerning proliferative, meiosis-entry, follicle-formation and embryo-generation properties of the OSCs. Furthermore, the identification of a small subpopulation of haploid cells suggests that, in certain circumstances, these cells have the capacity to spontaneously undergo meiosis [11].

However, the desired translation of results from mouse model to human fertility applicative programs is still far away for several obstacles mainly of ethical nature. The direct adoption of these procedures in women with ovarian insufficiency will have to wait as additional studies are needed to investigate their suitability and safety in humans.

## 6. A Perspective Utilization of Human OSCs for Women Infertility

Today, the female infertility is an emerging disorder with incidence as high as up to near 80 million women worldwide and is suspected to further increase in the future, also in relation to multiple endocrine dysfunctions promoted by lifestyle habits and environmental hazards [40].

Female infertility is pathogenically related not only to a number of conditions as uterine malformations, tubal obstruction, endocrine ovulatory disorders and cervical factors but also to other disorders such as X-chromosome linked disorders, autoimmunity related endometriosis, ovarian cysts and annessiectomy for benign or malignant ovary tumors inducing pre-mature exhaustion of the oocyte pool [41]. Women suffering from premature ovarian failure (POF) or in perimenopause period may ameliorate physiology of immune, circulatory and metabolic systems through diet, exercise and healthcare implementation which can indirectly provide a suitable micro-environment to improve ovarian function [42].

However, these precautions are not always functional, and women affected by ovarian defective physiology are inclined to undergo specific therapeutic options including oocyte or embryo donation.

In this context the functional properties of the OSCs to restore the follicle pool are to be considered a fruitful alternative approach not only to delay female genital aging and/or relieve the symptoms associated with perimenopausal phase but also as a possibility to achieve a physiologic pregnancy through both the differentiation and fertilization of these cells.

Another interesting application of fertility preservation by OSCs is to be also envisaged in women undergoing cancer treatments during their fertile age. Before neoadjuvant or adjuvant chemotherapy protocols as well as before ratiotherapy treatments, these patients are usually scheduled for recovering and freezing their eggs after hormone stimulation. However, although functional in successfully providing pregnancies, this procedure is not to be considered fully safe in particular in both pre-puberal and fertile women suffering from hormone-dependent tumors such as breast and gynecological cancers. In these patients, hormone stimulation to regulate ovulatory cycles and prepare for oocyte recruitment is potentially dangerous since specific molecular pathways of hormonal receptors are highly sensitive to the excess of estrogen bioavailability both in cancer cells and in other tissues expressing those receptors. Therefore, a safe alternative to avoid hormone stimulation is provided by the recovering of OSCs from the ovary cortex, subsequent differentiation in vitro on oocyte-like cells, selection of the most viable oocytes, and cryopreservation of a number of these mature cells. This procedure can reset the adjunctive cancerogenic risk of hormone stimulation in female patients with hormone-dependent neoplasms.

On the other hand, this procedure would also overcome the transplantation of autologous ovary cortex which is sometimes used in several oncologic centers to preserve fertility in women undergoing cancer treatments [42]. In fact, while the implant of the defrozen cortex fragment after cancer remission may be unsuccessful to promote the oocyte maturation in relation to the unknown number of follicles included in the cortex biopsy, the recruitment of a cohort of mature oocytes would offer the advantage of selecting the most suitable eggs for the fertilization procedure.

Therefore, the OSCs for fertility preservation in cancer patients after treatment undoubtedly lead to significant benefits for the lower risk of developing other tumors.

## 7. Conclusions

In conclusion, major progress has been made in the OSC field and it is time to arrive to a consensus regarding their existence rather than maintaining skepticism by focusing on technical issues. Besides the potential, future translation of this new knowledge into female infertility treatment, results from OSC studies open new possibilities for the menopause delay in the control of associated morbidities such as osteoporosis or restoring the ovarian function in cancer survivors. Based on preliminary observations by Tilly’s group in mice, mitotically active OSCs have been definitely detected in human ovarian cortex and experimental work by different groups of researchers including our own provide evidence that these cells are capable of generating oocytes during the women lifespan.

Bhartiya and co-workers also demonstrated that VSELs located in the OSE naturally differentiate into oocyte-like structures while we provided further evidence of the occurrence of OSCs in the ovarian cortex of non-menopausal and menopausal women and the ability of these cells to differentiate to form mature OLCs in vitro and acquire haploid chromosomal content.

Horan and Williams, in their comprehensive review, have recently explained the contrasting opinions on the OSC existence and on the potential use of these cells for fertility restoration.

Although today there is a wide debate on the physiological role of the OSCs in adult mammalian ovaries and the regulatory networks that control OSC self-renewal capacity versus differentiation into oocytes, additional studies are needed to demonstrate their suitability in humans and clarify the scientific skepticism on the OSC potential to restore the oogenesis.

## References

[1] Virant-Klun I. (2015). Postnatal oogenesis in humans: A review of recent findings. Stem Cells Cloning.

[2] Yazdekhasti H., Rajabi Z., Parvari S., Abbasi M. (2016). Used protocol for isolation and propagation of ovarian stem cells, different cells with different traits. J. Ovarian Res..

[3] Woods D.C., Tilly J.L. (2013). An evolutionary perspective on adult female germline stem cell function from flies to humans. Seminars in Reproductive Medicine.

[4] Bhartiya D. (2017). Letter to editor: Rejuvenate eggs or regenerate ovary?. Mol. Cell Endocrinol..

[5] Johnson J., Canning J., Kaneko T., Pru J.K., Tilly J.L. (2004). Germline stem cells and follicular renewal in the postnatal mammalian ovary. Nature.

[6] Zuckerman S. (1951). The number of oocytes in the mature ovary. Rec. Prog. Horm. Res..

[7] Johnson J., Bagley J., Skaznik-Wikiel M., Lee H., Adams G.B., Niikura Y., Tschudy K.S., Tilly J.C., Cortes M.L., Forkert R. (2005). Oocyte generation in adult mammalian ovaries by putative germ cells in bone marrow and peripheral blood. Cell.

[8] Virant-Klun I., Stimpfel M., Cvjeticanin B., Vrtacnik-Bokal E., Skutella T. (2013). Small SSEA-4-positive cells from human ovarian cell cultures: Related to embryonic stem cells and germinal lineage?. J. Ovarian Res..

[9] Virant-Klun I., Rozman P., Cvjeticanin B., Vrtacnik-Bokal E., Novakovic S., Rülicke T., Dovc P., Meden-Vrtovec H. (2009). Parthe-nogenetic embryo-like structures in the human ovarian surface epithelium cell culture in postmenopausal women with no naturally present follicles and oocytes. Stem Cells Dev..

[10] Stimpfel M., Skutella T., Cvjeticanin B., Meznaric M., Dovc P., Novakovic P., Cerkovnik P., Vrtacnik-Bokal E., Virant-Klun I. (2013). Isolation, characterization and differentiation of cells expressing pluripotent/multipotent markers from adult human ovaries. Cell Tissue Res..

[11] White Y.A.R., Woods D.C., Takai Y., Ishihara O., Seki H., Tilly J.L. (2012). Oocyte formation by mitotically active germ cells purified from ovaries of reproductive-age women. Nat. Med..

[12] Silvestris E., D’oronzo S., Cafforio P., D’Amato G., Loverro G. (2015). Perspective in infertility the ovarian stem cells. J. Ovarian Res..

[13] Silvestris E., Cafforio P., D’Oronzo S., Felici C., Silvestris F., Loverro G. (2018). In vitro differentiation of human oocyte-like cells from oogonial stem cells: Single-cell isolation and molecular characterization. Hum. Reprod..

[14] Bhartiya D., Patel H., Sharma D. (2019). Heterogeneity of Stem Cells in the Ovary.

[15] Woods D.C., Tilly J.L. (2015). Woods and Tilly reply. Nat. Med..

[16] Horan C.J., Williams S.A. (2017). Oocyte stem cells: Fact or fantasy?. Reproduction.

[17] Woods D.C., Tilly J.L. (2013). Isolation, characterization and propagation of mitotically active germ cells from adult mouse and human ovaries. Nat. Protoc..

[18] Virant-Klun I., Zech N., Rozman P., Vogler A., Cvjeticanin B., Klemenc P., Maličev E., Meden-Vrtovec H. (2008). Putative stem cells with an embryonic character isolated from the ovarian surface epithelium of women with no naturally present follicles and oocytes. Differentiation.

[19] Bhartiya D. (2015). Ovarian stem cells are always accompanied by very small embryonic-like stem cells in adult mammalian ovary. J. Ovarian Res..

[20] Ratajczak M.Z., Ratajczak J., Suszynska M., Miller D.M., Kucia M., Shin D.M. (2017). A novel view of the adult stem cell compartment from the perspective of a quiescent population of very small embryonic-like stem cells. Circ. Res..

[21] Ganguly R., Metkari S., Bhartiya D. (2017). Dynamics of bone marrow VSELs and HSCs in response to treatment with gonadotropin and steroid hormones, during pregnancy and evidence to support their asymmetric/symmetric cell divisions. Stem Cells Rev. Rep..

[22] Bhartiya D., Parte S., Patel H., Anand S., Sriraman K., Gunjal P., Mariusz R. (2014). Pluripotent very small embryonic-like stem cells in adult mammalian gonads. Adult Stem Cell Therapies: Alternatives to Plasticity, Stem Cell Biology and Regenerative Medicine.

[23] Patel H., Bhartiya D., Parte S., Gunjal P., Yedurkar S., Bhatt M. (2013). Follicle stimulating hormone modulates ovarian stem cells through alternately spliced receptor variant FSH-R3. J. Ovarian Res..

[24] Ding X., Liu G., Xu B., Wu C., Hui N., Ni X., Wang J., Du M., Teng X., Wu J. (2016). Human GV oocytes generated by mitotically active germ cells obtained from follicular aspirates. Sci. Rep..

[25] Woods D.C., Tilly J.L., Sanders S. (2013). Germline stem cells in adult mammalian ovaries. Ten Critical Topics in Reproductive Medicine.

[26] Cha K.Y., Chian R.C. (1998). Maturation in vitro of immature human oocytes for clinical use. Hum. Reprod. Update.

[27] Akahori T., Woods D.C., Tilly L.J. (2019). Female Fertility Preservation through Stem Cell-based Ovarian Tissue Reconstitution in Vitro and Ovarian Regeneration in Vivo. Clin. Med. Insights Reprod. Health.

[28] Hübner K., Fuhrmann G., Christenson L.K., Kehler J., Reinbold R., De La Fuente R., Wood J., Strauss J.F., Boiani M., Schöler H.R. (2003). Derivation of oocytes from mouse embryonic stem cells. Science.

[29] Woods D.C., White Y.A.R., Niikura Y., Kiatpongsan S., Lee H.J., Tilly J.L. (2013). Embryonic stem cell-derived granulosa cells participate in follicle formation in vitro and in vivo. Reprod. Sci..

[30] 30 Truman A.M., Tilly J.L., Woods D.C. (2017). Ovarian regeneration: The potential for stem cell contribution in the postnatal ovary to sustained endocrine function. Mol. Cell Endocrinol..

[31] Fakih M.H., El Shmoury M., Szeptycki J., de la Cruz D.B., Lux C., Verjee S., Burgess C.M., Cohn G.M., Casper R.F. (2015). The AUGMENTSM treatment: Physician reported outcomes of the initial global patient experience. JFIV Reprod. Med. Genet..

[32] Yamashiro C., Sasaki K., Yabuta Y., Kojima Y., Nakamura T., Okamoto I., Yokobayashi S., Murase Y., Ishikura Y., Shirane K. (2018). Generation of human oogonia from induced pluripotent stem cells. Science.

[33] Hermann B.P., Sukhwani M., Winkler F., Pascarella J.N., Peters K.A., Sheng Y., Valli H., Rodriguez M., Ezzelarab M., Dargo G. (2012). Spermatogonial stem cell transplantation into rhesus testes regenerates spermatogenesis producing functional sperm. Cell Stem Cell.

[34] Sato T., Katagiri K., Gohbara A., Inoue K., Ogonuki N., Ogura A., Kubota Y., Ogawa T. (2011). In vitro production of functional sperm in cultured neonatal mouse testes. Nature.

[35] Zheng W., Zhang H., Gorre N., Risal S., Shen Y., Liu K. (2014). Two classes of ovarian primordial follicles exhibit distinct developmental dynamics and physiological functions. Hum. Mol. Gen..

[36] Shim H.J., Lee E.M., Nguyen L.D., Shim J., Song Y.H. (2014). High-dose irradiation induces cell cycle arrest, apoptosis, and developmental defects during Drosophila oogenesis. PLoS ONE.

[37] Bui H.T., Van Thuan N., Kwon D.N., Choi Y.J., Kang M.H., Han J.W., Kim T., Kim J.H. (2014). Identification and characterization of putative stem cells in the adult pig ovary. Development.

[38] Telfer E.E. (2004). Germline stem cells in the postnatal mammalian ovary: A phenomenon of prosimian primates and mice?. Reprod. Biol. Endocrinol..

[39] Telfer E.E., Albertini D.F. (2012). The quest for human ovarian stem cells. Nat. Med..

[40] Nattiv A., Loucks A.B., Manore M.M., Sanbom C.F., Sundgot-Borgen J., Warren M.P. (2007). American College of Sports Medicine position stand. The female athlete triad. Med. Sci. Sports Exerc..

[41] Practice Committee of American Society for Reproductive Medicine (2012). Diagnostic evaluation of the infertile female: A committee opinion. Fertil. Steril..

[42] Haifeng Y., Tuochen Z., Wei L., Xiaoyan L., Xinxin F., Yaoqi H., Hu C., Li J., Huang J., Liu Z. (2017). Ovarian Stem Cell Nests in Reproduction and Ovarian Aging. Cell Physiol. Biochem..

